# Effect of coronary artery bypass grafting surgery with a pump on cerebral blood flow in high-risk patients

**DOI:** 10.1186/cc14551

**Published:** 2015-03-16

**Authors:** R Juliana, G Ferreira, L Camara, S Zefferino, D Azevedo, R Groehs, M Lima, R Nogueira, E Bor-Seng-Shu, E Osawa, J Jardim, J Almeida, F Galas, L Hajjar

**Affiliations:** 1 Heart Institute, São Paulo, Brazil

## Introduction

Coronary artery bypass grafting (CABG) surgery usually improves myocardial contractility, reducing cardiovascular events. However, it is a high-risk procedure associated with significant neurological complications, including stroke, delirium and cognitive impairment. The pathophysiology of these complications is not very well known, and may include low flow state after surgery, low cardiac output, embolism and reperfusion lesion. The aim of this study is to prospectively evaluate the cerebral hemodynamics through transcranial color and spectral Doppler sonography in high-risk patients undergoing cardiac surgery with a pump.

## Methods

This was a prospective, single-center study, performed at the Heart Institute from University of São Paulo. From May to November 2014 we included 35 patients in the study, aged older than 18 years old, submitted to CABG with a pump, with EuroSCORE higher than 6 or left ventricular ejection fraction lower than 40%. Transcranial color and spectral Doppler sonography was performed 48 hours before surgery (T0), 7 days (T1) and 6 months after surgery (T2). We used a probe of 2.5 to 2 MHz (Doppler Box DWL/Compumedics, Singen, Germany). All recordings were taken with the patient in a supine position. We measured the middle cerebral artery mean flow velocity and pulsatility index. The end-expiratory pressure of CO_2_ (PETCO_2_) was measured with infrared capnography attached to a face mask. Blood pressure, hematocrit and axillary temperature was also recorded.

## Results

The mean age of patients was 64 years; most patients were male (74%). Middle cerebral artery mean flow velocity increased significantly after cardiac surgery. It was 53.89 ± 17.23 m/second at T0, 61.48 ± 15.18 m/second at T1 and 59.27 ± 16.12 m/second at T2 (*P *= 0.029). The pulsatility index was similar at all time points (0.88 ± 0.25 at T0, 0.85 ± 0.24 at T1 and 0.91 ± 0.25 at T2, *P *= 0.146). There was a significant difference in the levels of hemoglobin (13.19 ± g/dl 1.97 at T0 and 9.64 ± 1.48 g/dl at T1, *P *= 0.002). However, this difference was not maintained at T2 (12.7 ± 2.02 g/dl at T2, *P *= 0.252). There were no differences regarding PETCO_2_ at the time points.

## Conclusion

After cardiac surgery with a pump in high-risk patients, improvement of cerebral hemodynamic occurs, perhaps due to the optimization of cardiovascular function. These findings must be better investigated.

